# Clay Nanotube Immobilization on Animal Hair for Sustained Anti-Lice Protection

**DOI:** 10.3390/pharmaceutics13091477

**Published:** 2021-09-15

**Authors:** Naureen Rahman, Faith Hannah Scott, Yuri Lvov, Anna Stavitskaya, Farida Akhatova, Svetlana Konnova, Gӧlnur Fakhrullina, Rawil Fakhrullin

**Affiliations:** 1 Institute for Micromanufacturing and Biomedical Engineering Program, Louisiana Tech University, Ruston, LA 71272, USA; nra015@latech.edu (N.R.); fhs002@email.latech.edu (F.H.S.); 2 Department of Physical and Colloid Chemistry, Gubkin University, Leninsky Pr. 65, 119991 Moscow, Russia; stavitsko@mail.ru; 3 Institute of Fundamental Medicine and Biology, Kazan Federal University, Kreml urami 18, 420008 Kazan, Republic of Tatarstan, Russia; akhatovaf@gmail.com (F.A.); Svetaka14@gmail.com (S.K.); GIFahrullina@kpfu.ru (G.F.)

**Keywords:** hair surface engineering, topical drug administration, antiparasitic formulations, halloysite, self-assembly, capybara, guinea pig

## Abstract

Topical administration of drugs is required for the treatment of parasitic diseases and insect infestations; therefore, fabrication of nanoscale drug carriers for effective insecticide topical delivery is needed. Here we report the enhanced immobilization of halloysite tubule nanoclay onto semiaquatic capybaras which have hydrophobic hair surfaces as compared to their close relatives, land-dwelling guinea pigs, and other agricultural livestock. The hair surface of mammals varies in hydrophobicity having a cortex surrounded by cuticles. Spontaneous 1–2 µm thick halloysite hair coverages on the semi-aquatic rodent capybara, non-aquatic rodent guinea pig, and farm goats were compared. The best coating was found for capybara due to the elevated 5 wt% wax content. As a result, we suggest hair pretreatment with diluted wax for enhanced nanoclay adsorption. The formation of a stable goat hair coverage with a 2–3 µm halloysite layer loaded with permethrin insecticide allowed for long-lasting anti-parasitic protection, enduring multiple rain wettings and washings. We expect that our technology will find applications in animal parasitosis protection and may be extended to prolonged human anti-lice treatment.

## 1. Introduction

Mammal skin is covered with proteinaceous hair fibers, which have a variety of functions including sexual recognition, skin protection, heat conservation, and waterproofing. Unfortunately, hair provides a dwelling place for numerous parasites, including arthropods. For example, mammals are known to host approximately 5000 species of lice and ticks populating fur and other hair-like filamentous skin coatings. Apart from the direct damage incurred by sucking and chewing hair arthropods, these parasites also transmit several pathogens to humans, pets, and wild animals. In particular, the livestock industry is greatly affected by infestations of lice. Heavily infested animals will suffer from retarded growth and weight gain, and these poorly nourished animals will experience an increased mortality rate, leading to a loss in the production of meat and milk [1]. Unlike in humans, where modern hygiene standards have significantly reduced lice infestation, the treatment of farm animals is more troublesome. The isolation of the infected individuals or frequent reapplications of insecticides is not always effective, especially when a mass infestation occurs. Most of the commercially available pour-on or spot-on processes of anti-lice treatments have a rainfast time of about 6 h (for example, ABACARE 1% *w*/*v* Pour-on Solution: J&M, Ireland). Ivermectin or pyrethroid-based insecticides such as permethrin are lipophilic which implies that rain can significantly cut down the protection periods and thus lead to under-dosing and reduced insecticide effects. The topical delivery of insecticides requires a sustainable application as a therapeutic carrier system. Therefore, novel tools are needed to secure effective and long-lasting insecticide deposition onto animals’ hair.

Clay minerals, such as montmorillonite and sepiolite, have been effectively used as pharmaceutical excipients for oral and topical drug delivery systems [2]. Likewise, halloysite clay nanotubes (Al_2_Si_2_O_5_(OH)_4_) may act as a carrier for the anti-lice drug delivery and readily attach to the hair [3,4]. Application of halloysite self-assembly develops a robust and uniform coating for hair surface engineering, which we pioneered [3]. Water-insoluble anti-lice drugs such as permethrin and ivermectin are especially difficult for topical body placement but can be encapsulated into the halloysite clay tube’s lumens followed by a spontaneous self-assembly deposition of drug-loaded clays onto hair from aqueous suspensions. Moreover, halloysite-loaded drug formulation can be stored in dry forms for extended periods, making it more accessible and easier to handle in field conditions. Halloysite self-assembles on the hair with the short-time application of its aqueous colloids leaving a 2–3 µm thick uniform coating [4]. After being loaded with drugs, halloysite provides a slow 5–10-day release allowing for a sustained topical anti-parasitic treatment. Halloysite is a naturally occurring tubule aluminosilicate clay mineral with an external diameter of 50–60 nm, an inner diameter of 10–15 nm, and a length within 0.5–1 μm.

Halloysite has opposite surface charges; the external surface is composed of Si−O−Si groups posing negative charge whereas the inner surface consists of a gibbsite-like array of Al−OH groups posing positive charge in aqueous solution with pH 3.3–8.5 [5,6,7]. As a result, halloysite is suitable for the encapsulation of negatively charged drugs, proteins, and dyes inside the positive lumen (which is the simplest and most efficient method), and the cationic molecules can be tailored to the outer surfaces of the nanotubes [5,6]. Hydrophobic drugs have also been encapsulated into halloysite lumens [8,9]. The siloxane groups located on the exterior surface of the tubes with low contents of hydroxyl groups render the outer layer partially hydrophobic [10]. These partially lipophilic characteristics help the self-assembly process of halloysite nano clay on hair. Hair cuticles are also naturally partially hydrophobic; therefore, they exhibit an enhanced affinity to halloysite nanotubes [3,11]. A hair follicle is comprised of a keratin cortex and at its center is protected by several layers of the cuticle [12]. The cuticle cells are covered by epicuticle, a monolayer of covalently 18-methyleicosanoic acid-lipid components contributing to the hydrophobicity of hair [13]. Although under the microscope all mammal hair fibers are structurally identical, their characteristics differ among living species [14]. The hair of aquatic and semi-aquatic animals is more water repellent than terrestrial species.

**Figure 1:**
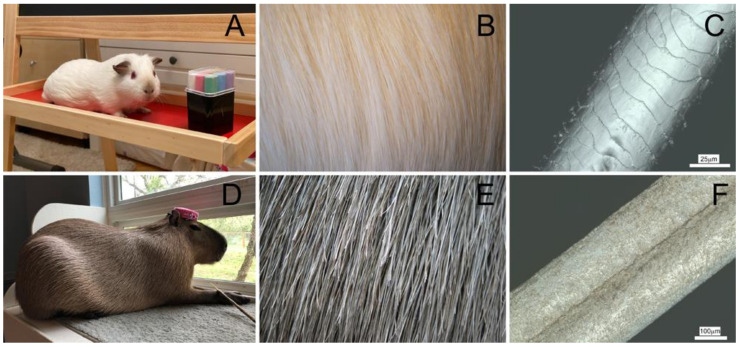
American breed guinea pig *C. porcellus* (**A**) is covered by a dense layer of insulating, thin, unwaxed hair (**B**), while the capybara *H. hydrochaeris* (**D**) skin is covered by long, thick, double-curved, and mechanically strong hair (**E**) aimed to protect the skin from scratches and make it water-resistant. The 3D confocal optical microscopy images of guinea pig (**C**) and capybara hair (**F**) demonstrate the fine structure and surface appearance of the hair cuticle.

In this paper, we demonstrate pristine halloysite nanotube coverage on animal fur checking different degrees of hair hydrophobicity due to higher wax content for aquatic species. We analyzed the hydrophobicity of the semi-aquatic rodent capybara (*Hydrochoerus hydrochaeris*) [15] and non-aquatic rodent guinea pig (*Cavia porcellus*) hairs and observed halloysite coating on them. Capybaras spend a significant portion of their life in water, and the presence of wax on the hair functions to provide water resistivity. Guinea pigs lack this wax coating as this rodent is systematically similar but ecologically different than the capybara. The hair/fur of these systematically closely related animals existing in different habitats have evolved different surface wettability. The typical photographs of the appearance of capybara and guinea pig and their hair are shown in Figure 1. Capybara hair contains wax and has been used recently as a bioindicator to investigate the metals distribution in ecotoxicological studies [16].

Permethrin is an effective drug that eradicates arthropod ectoparasites such as lice, mites, and horn flies; therefore, it is commonly used as a pour-on treatment for cattle [17]. Permethrin is also used for human scalp applications as a treatment for infestations of bloodsucking lice [18]. A single administration of permethrin is not sufficient for extended antiparasitic protection. In addition, weather conditions (rainfalls), animals’ grooming, and swimming facilitate the removal of the insecticide from the fur. As a result, numerous reapplications of permethrin are needed, and this increases costs and may be harmful to the animal [19]. Alternatively, encapsulation of permethrin into semi-permeable nanocontainers may extend the drug release time and avoid the need for reapplications while reducing the unwanted side effects. Biocompatible halloysite nanotubes are excellent natural nanocontainers for loading and sustained release of various drugs. Moreover, the dried formulation based on halloysite can be kept on a shelf for years and dispersed in water immediately before application [3,4,20]. As shown previously for human hair, once the loaded halloysite dispersion is applied to hair, the clay coating remains stable for 5–6 shampoo washes [3], facilitating the slow drug release. A single and long-lasting treatment of the nano clay-loaded antiparasitic formulation is cost-effective and saves time as compared to other administration routes requiring repeated applications.

Since permethrin is being used currently to treat goat lice [17], the efficacy of halloysite-drug composite was observed on Trichodectidae chewing lice *Damalinia caprae* (alternatively known as *Bovicola caprae*) hosted by farm Boer goats (*Capra aegagrus hircus*). The coating was performed by treating animal hair with 5 wt% nano clay dispersion in water followed by drying which resulted in a 2–3 µm thick coating. An analysis of the coating efficiency with different hydrophobicity of fur (waxed-fur capybara, guinea pig, and goat) allowed for optimization of the halloysite coating formulations for the drug-loaded nanotube targeting lice. The pediculicidal activity of the composite against lice was observed with sequential treated hair washing and reintroduction of new batches of lice. A wax pre-treatment method was also used for the improvement of halloysite nanotube goat coating, similar to numerous cosmetic hair products such as conditioner, hair shiner, etc., that include wax or a wax-like substance [18,21]). We suggested permethrin-nanotube coating formulations which can be implemented in veterinary antiparasitic treatment and may be used to treat psoriasis and fungal infections; a similar formulation may be considered for humans.

## 2. Materials and Methods

### 2.1. Halloysite Aqueous Dispersion for Hair Coating

Halloysite nanotubes (HNTs) were provided by Applied Minerals Inc., Brooklyn, NY, USA. 80% of the tubes’ length falls in the range of 730 ± 200 µm while 70% of the tubes’ external diameters are 60 ± 10 nm. 500 mg of HNTs was added in 10 mL of DI water (5% *w*/*v*) and the dispersion was sonicated for 5 min using a Branson 1800 ultrasonic bath (Danbury, CT, USA) and then stirred for 15 min.

### 2.2. Coating of the Animal Hair with Halloysite Dispersion

Capybara (*H. hydrochaeris*) hair samples were obtained from a four-year-old female. American breed guinea pig (*C. porcellus*) samples were obtained from several one to four-year-old female animals. Both capybaras and guinea pigs were kept indoors as domestic pets and provided standard food, care, and entertainment. Boer goat hair was taken from two-year-old goats from a farm located in Homer, Louisiana. Hair samples were obtained during regular grooming, which did not inflict any stress or suffering on the animals. All the hair samples were kept in closed petri dishes at 24 °C and humidity of 32%. To coat with halloysite dispersion, the hair samples were chopped to 4–5 cm. Two-hundred milligrams of hair was taken in a petri dish for each of the coating experiments and 0.8 mL of aqueous 5 wt% halloysite dispersion was dropped on the hair samples, hand massaged for one minute, and allowed to rest for 5 min. To remove the excess halloysites, the hair was rinsed with tap water and air-dried. To remove the natural wax layer from capybara hair, it was treated with acetone (ACS-Labchem, Zelienople, PA, USA). On a glass petri dish, 200 mg of hair was submerged in 10 mL of acetone and allowed to rest for 10 min. The treated hair was rinsed with tap water and unwaxed capybara hair was achieved.

### 2.3. Hair Surface Study

The hair coverage was visualized using scanning electron microscopy (SEM, HITACHI S-4800 FESEM, Tokyo, Japan) and 3D laser scanning microscopy (VK-X150 confocal microscope, Keyence, Osaka, Japan). With SEM, the deposition could be seen with detail while the sample was coated with 15 nm gold sputtering. The samples were also imaged without any additional coating, and the coating height (Z-scale) can be obtained in a native state with 3D laser scanning microscopy. The numerical surface structure evolution, as well as width and diameter, were also obtained from the laser scanning microscope [22]. Hair cut in 5 cm increments was sampled and positioned diagonally on the glass slides, and the images were taken in superfine mode.

Atomic force microscopy (AFM) images were obtained using a Dimension Icon microscope (Bruker, Elmsford, NY, USA) equipped with Scan Asyst-Air (Bruker) probes (tip radius 2 nm, nominal length 115 µm, radius 2 nm, spring constant 0.4 nm^−1^) for surface topography. Raw data were analyzed with Nanoscope Analysis software (Bruker, v 1.7, Elmsford, NY, USA), as described in a previous report [23]. Hair segments were attached to regular microscopy slides with an adhesive type and then imaged in the air under ambient temperature and humidity in Peak Force Tapping QNM mode. To minimize the surface curvature interference, we imaged the top areas along the hair shaft.

### 2.4. Thermogravimetry of Pristine and Unwaxed Capybara Hair

Two pieces of equal lengths of pristine and unwaxed capybara hair were chopped so they could fit the pan and run through thermogravimetric analysis (TGA) (QA Instruments Q50 TGA - New Castle, Mettler Toledo, Wilmington, DE, USA). Using 0.5–0.6 mg of pristine hair, the wax content was analyzed by comparing the weight loss difference at 30–600 °C with a heating rate of 10 °C/min.

### 2.5. Hydrophobicity Characterization of Hair Specimens

To check the hydrophobicity of capybara, guinea pig hair, and HNT samples, water wettability of the samples was measured using a video-based contact angle meter (Contact Angle System OCA-FD Scientific Corp., Bethpage, NY, USA) at 22 ± 1 °C and 32 ± 5% RH. A droplet of 5 ± 0.5 µL DI water was put on the samples surfaces and recorded by a CCD camera. Three measurements were carried out on each of the samples to eliminate any error.

### 2.6. Loading of Halloysite Nanotubes with Permethrin

The anti-lice drug permethrin is a poorly water-soluble synthetic pyrethroid that is highly soluble in ethanol [24]. When dissolved in the solvent, the drug can be easily absorbed by halloysite nanotubes with continuous stirring and vacuuming. Borrego-Sánchez et al. studied its interaction with low aqueous soluble antiparasitic drugs with the other clay materials [2,25]. One hundred and fifty milligrams of permethrin (Sigma Aldrich, St. Louis, MO, USA) was dissolved in 3 mL of 95% ethanol (Sigma Aldrich) to obtain a saturated solution (50 mg/mL). Seventy-five milligrams of halloysite was added to this solution and mixed to obtain a homogeneous suspension (HNT/drug content 1:2). The solution was ultrasonicated for 5 min, vacuumed, and stirred simultaneously overnight to ensure maximum loading. The sample was then washed once with ethanol to remove the excess drug. The washing was done to analyze how much drug is encapsulated in the tubes and to analyze the efficacy of the loaded drug on animal parasites. Drug loading efficiency was determined using TGA over the temperature range 30–600 °C [26].

### 2.7. Surface Charge and Colloidal Stability Assessment of Halloysite

Permethrin and drug-loaded and unloaded halloysite dispersions were made in DI water at pH 7 to carry out surface potential measurements. To determine the stability of the aqueous solutions, the samples were stored at room temperature for 3 days while zeta potentials were measured every day with the Brookhaven Zeta-Plus instrument (Brookhaven, Holtsville, NY, USA) [27,28].

### 2.8. Drug Release Kinetics Evaluation

For the release kinetics study, drug release was observed in ethanol to demonstrate maximum release in the solvent for 48 h. Sixteen milligrams of halloysite-drug composites were added to 20 mL of 95% ethanol at 24 °C and were stirred using a magnetic stirrer at 200 rpm. One milliliter of each sample was collected at regular intervals and centrifuged at 5000 rpm for 5 min for UV-vis spectrophotometer (Agilent Chemstation UV−Vis, Shanghai, China) analysis. The analysis was carried out by setting the absorbance mode at λ_max_ 227 nm with 800 nm background correction. One milliliter of the fresh solvent was replenished to the release medium with the re-suspended powder that was obtained as sediment after the centrifugation so that the concentration always remained the same in the sink.

### 2.9. In Vitro Goat D. caprae Lice Eradication Bioassay

The chewing goat lice *D. caprae* used in this study were collected from infested Boer goats. Food for the lice was prepared by collecting flakes of skin that had detached from the goat. Young adult lice in good condition were collected by hand from infested donor goats and placed in glass petri dishes. Since permethrin is soluble in ethanol, each specimen, HNTs, and HNTs-drug composite samples for lice experiments on hair were prepared with 30% ethanol. On a petri dish, 0.80 mL of each formulation (Table 1, Table 2 and Table 3) were dropped on 0.2 g (4–5 cm long) of goat hair. The hair sample was gently massaged until the entire sample was made damp with the solution. In the lab, glass vial tubes were filled with skin flakes and 200 mg of coated hair, and batches of 10 lice were placed in each of the tubes. The tubes were placed in a hood maintaining 72% humidity and 37 ± 2 °C, in darkness. Each batch of lice was observed every hour for mortality. Lice mobility was observed via movements of the antennas and legs. The dead lice showed immobility with shrunken abdomens and desiccated bodies, and they were removed after detection. At least three sets of trials were performed for the hair tuft test.

### 2.10. Hair Pretreatment with Wax to Maximize Halloysite Coverage

To enhance the halloysite coating, goat hair was pretreated with carnauba wax (Sigma Aldrich, St. Louis, MO, USA). Ten milligrams of paraffin wax (Sigma Aldrich) was melted in 10 mL of 10% acetic acid (Sigma Aldrich) (0.10 wt% wax) by ultrasonication and heated at 85 °C. With a dropper, 0.8 mL of wax solution was dropped on 0.2 g of hair and gently massaged. Halloysite dispersion was used to coat the hair as mentioned before.

## 3. Results and Discussion

### 3.1. Surface Texture of Halloysite Coated Hair Cuticles

**Scheme 1:**
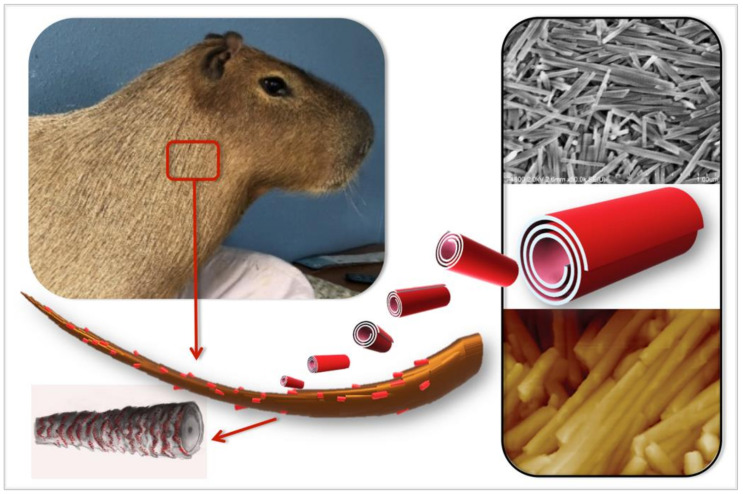
Self-assembly of halloysite clay nanotube on capybara hair. Nanotubes mostly accumulated near the cuticle edges of hair surface by a self-assembly process.

We demonstrate the principles of our approach of animal hair (fur) surface engineering in Scheme 1, where the deposition of halloysite onto capybara hair is depicted. Capybaras, the world’s largest rodents from South America, are becoming more popular as zoo animals [14] and even pets, thus requiring special attention for anti-parasite treatment. This is why we have chosen the capybara hair for detailed investigation.

**Figure 2:**
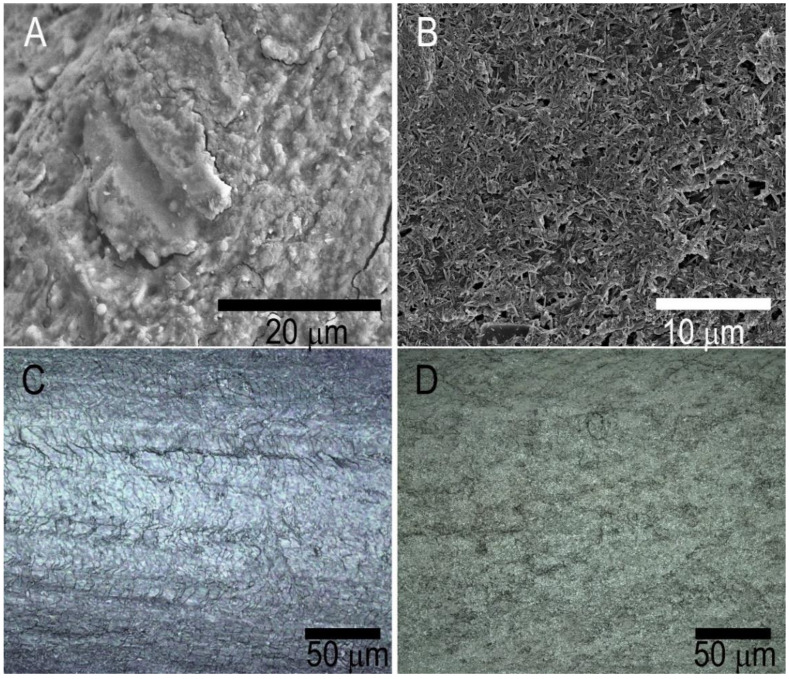
Self-assembly of halloysite clay nanotubes on capybara hair surface: SEM and 3D confocal optical microscopy images of the pristine capybara hair (**A**,**C**) and the surface is completely covered with HNTs (**B**,**D**).

We obtained SEM and 3D scanning confocal microscopy images of the pristine and acetone-treated (unwaxed) capybara hair samples (Figure 2). Capybara hair visually consists of two adjacent keratin filaments, forming a valley where the filaments are attached. The hair is hydrophobic due to the presence of the wax layer, which is required to render the hair surface more water-resistant. Although this aspect of capybara zoology was not studied thus far, to the best of our knowledge, we assume that hair in capybaras is not needed for thermoregulation. Like other aquatic mammals, a layer of subcutaneous fat protects from low temperatures. However, water-repellant hair is needed for better removal of water droplets once the animal is out of water. As we noticed, this wax layer on the hair can be completely coated with evenly distributed halloysite nanotubes, deposited following a previously established protocol [3]. This coating is considerably denser and thicker than prior reports of nanoclay coatings for human hair [3,4]. In Figure S1 we show the electron micrographs of acetone-treated capybara hair, where the wax layer was removed from the hair surface. Acetone dissolved hair wax, but it did incur damage to keratin protein cuticles located beneath the wax layer. Due to the absence of wax, the halloysite coating was less prominent and not evenly distributed, probably being hanged mostly in the remaining hydrophobic sites between the cuticles. Halloysite distribution of two types of capybara hair was also visualized with 3D laser scanning microscopy (Figure 2C,D). These images confirm the effective spatial deposition of the nanotubes onto the natural wax-covered capybara hair.

Capybara hair samples have diameters of 105 ± 3 μm, which includes a surface wax layer. After 5 min of exposure to 5 wt% halloysite dispersion, a 3.0 ± 0.8 μm thick coating was observed with the cross-section imaging (Figure S2A). We estimated the coating area population with the nanotubes as ca. 70%. The distribution of halloysite nanotubes was detected all over the pristine hair surface making a thin film layer on it which can be observed in Figure 2B. The thickness of the coating on the unwaxed hair was 2 ± 0.5 μm, (Figure S2B). Halloysites nanotubes were mostly targeting the cuticle areas of the hair surface. As a result, the coverage area decreased almost twice as compared to the wax-coated pristine capybara hair. These results indicate that the natural surface coatings, which are different from species to species, are important for hair surface engineering, and they should be taken into consideration.

**Figure 3:**
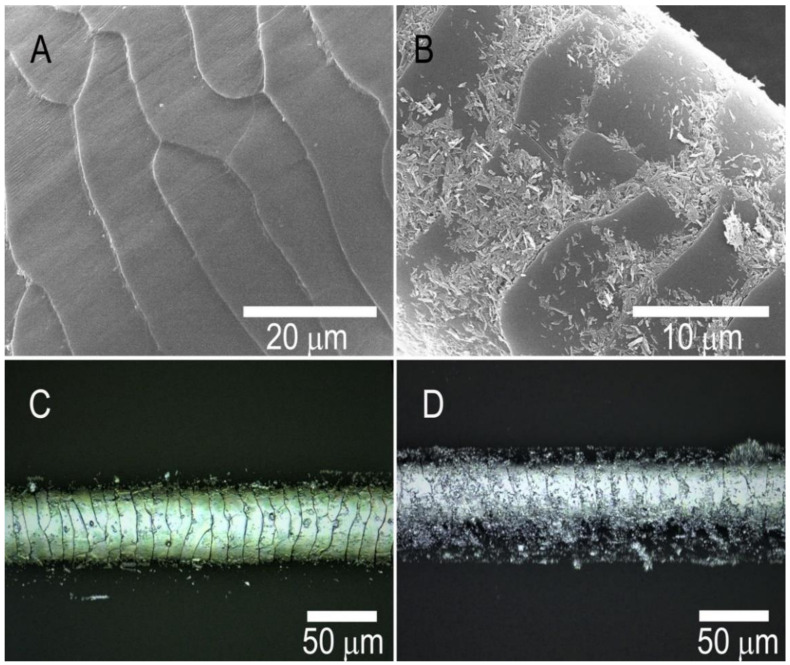
Self-assembly of the clay nanotubes on guinea pig hair surface: SEM and 3D confocal optical microscopy images of the pristine hair (**A**,**C**), and hair surface coated with halloysites where nanotubes are mostly accumulated near the cuticle openings (**B**,**D**).

Our first choice of species to compare capybara hair with were guinea pigs, South American animals domesticated by Native Americans, which have been used for millennia by humans as food, laboratory animals, and home pets. The primary reason to compare guinea pig hair with that of capybaras is that both species are closely related in zoological terms, but they live in completely different habitats (dry and wet). Figure 3 demonstrates the SEM images of halloysite-coated and uncoated adult American breed guinea pig hair samples. Unlike capybara hair, guinea pig hair is originally non-waxy, making it less hydrophobic (similar to human hair.) We noticed that the deposition of halloysite coating on guinea pig hair resulted in ca. 50% of the hair area coverage. The distribution of the nanotubes is predominantly near the cuticle edges, similarly to the unwaxed capybara hair, as shown in Figure 2.

Guinea pig hair samples have diameters of 64 ± 2 μm. Since guinea pig hair is naturally less hydrophobic than capybara hair, after exposure of 5 wt% halloysite on hair, 1.5 ± 0.3 μm coating was observed with cross-section imaging (Figure S3). Using 3D confocal measuring microscopy, we explored the surface texture parameters of pristine and nanoclay-coated capybara and guinea pig hair (Table 1). The squared mean height (Sq) of halloysite coated capybara pristine hair went much higher than unwaxed ones because of higher HNT deposition. With guinea pig hair Sq also did not go as high as HNTs coated capybara pristine hair since the deposition was less prominent. The maximum height value (Sz) also increased after deposition of HNT on capybara pristine hair. For comparison with another agricultural animal, we used horsehair coated with halloysite which resulted in a similar coating efficiency; the corresponding SEM and 3D confocal images are provided in Figures S4 and S5. The skewness (Ssk) evaluates the height distribution of the surface of hair which deviates slightly with regards to the mean profile line. The smaller values of autocorrelation length (Sal) of coated hair than untreated and uncoated hair suggest that the nanoparticle sizes are finer than the bare hair cuticles.

**Figure 4:**
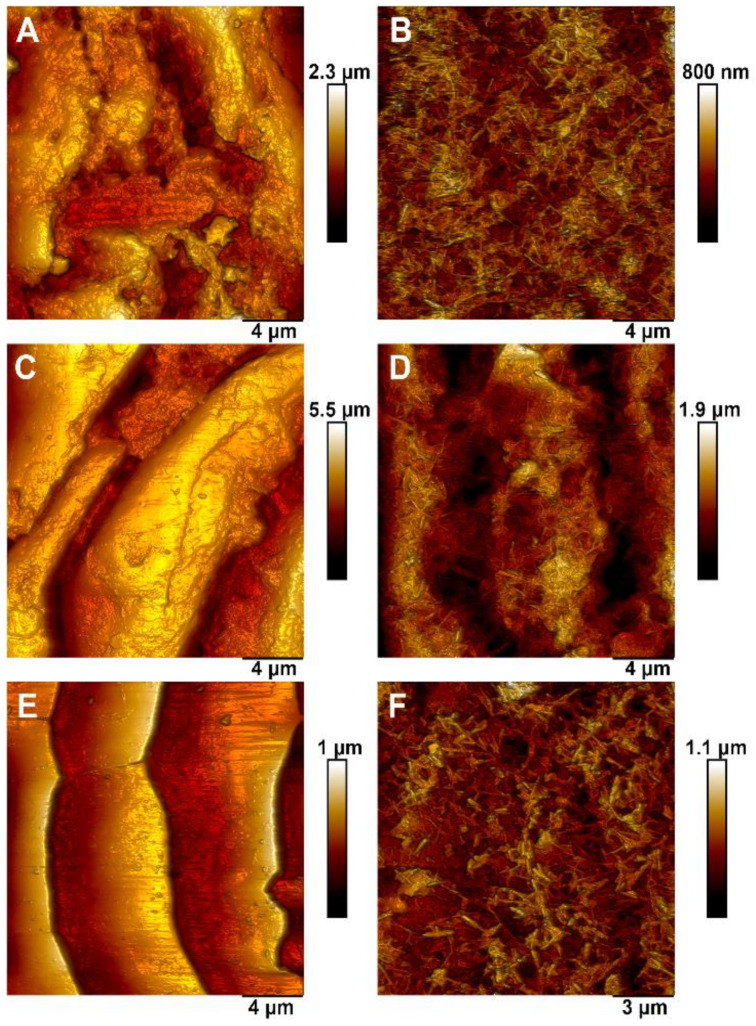
AFM topography images of capybara (**A**–**D**) and guinea pig (**E**,**F**) hair before and after coating with halloysite. Interestingly, on the nanoscale, the wax-free capybara hair demonstrated the increased cuticle roughness of both Ra (arithmetical mean roughness) and Rq (root mean squared roughness) which occurs apparently due to the removal of the smoothing wax layer.

For a more detailed characterization of the naturally wax-coated hair of capybara and wax-free guinea pig and the same samples after the nanoclay coating, we subjected the hair samples to AFM investigation, which allows for simultaneous imaging and mechanical properties mapping [29,30,31] The topography, non-specific adhesion, and Young’s modulus images of pristine unwaxed capybara hair compared to that of a guinea pig are given in Figure S6. One can see that the cuticle scales in capybara hair are fully covered by the uneven, thick wax layer, which, apart from making the surface non-uniform in height renders it uniform for tip adhesion and surface stiffness. On the contrary, in guinea pig hair the absence of a substantial wax coverage results in a periodic surface structure with clearly visible individual cuticles, having higher non-specific adhesion and lower stiffness at the edges, similarly to what was previously reported for human hair [22]. Further, we investigated the deposition of halloysite nanotubes onto the pristine and unwaxed capybara hair and guinea pig hair using AFM (Figure 4). The images of the pristine capybara hair have confirmed the formation of a dense wax layer (estimated earlier as 5 wt%) which completely covered the underlying keratin cuticle. Interestingly, the inter cuticle voids in non-waxed guinea pig hair exhibited higher non-specific adhesion and lower modulus, if compared with cuticle surfaces. In capybara hair, the individual cuticles could not be visualized due to the outermost wax, but the following halloysite coating was thicker and more uniform. The AFM-based mapping patterns of adhesion and modulus for capybara and guinea pig hair before and after coating with halloysite nanotubes are presented in Figure S7.

Nanomechanical properties, such as non-specific adhesion and Young’s modulus (obtained following the Derjaguin, Muller, and Toropov model) have also been investigated using AFM operating in Peak Force Tapping mode. The results (Table 2) demonstrate a higher non-specific adhesion in guinea pig cuticles, as well as significantly higher modulus. In capybara hair, the removal of the wax layer resulted in the decrease of adhesion, which we attribute to reduced hydrophobicity of the wax-free keratin. The modulus also increased for the wax-free capybara hair, which is softer than the underlying keratin layer. Therefore, the naturally waxed capybara hairs are smoother and softer.

### 3.2. Hair Water Contact Angle, Waxing, and Halloysite-Drug Loading

**Figure 5:**
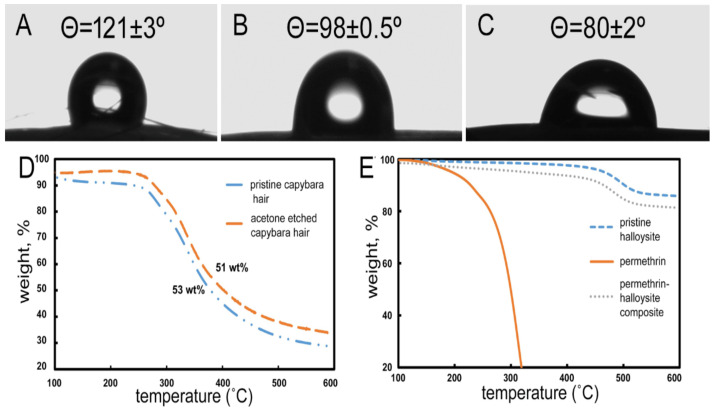
Water contact angle on pristine capybara hair (**A**), guinea pig (**B**), and acetone treated (unwaxed) capybara hair (**C**); thermogravimetric analysis of capybara pristine and acetone etched hair (**D**); and of permethrin, pristine halloysite, and permethrin-halloysite composite (**E**).

The deposition of hydrophobic material onto hair, which is a normal evolutionary development in certain amphibious mammals, decreases their wettability. In Figure 5A–C we demonstrate that the contact angle of capybara hair surfaces decreases with the wax removal and approaches that of the water-avoiding guinea pig. The elevated contact angle in pristine guinea pig hair, if compared with dewaxed capybara hair, is due to the lipophilic molecules always present in unwashed animal or human hair, whereas the capybara hair was treated with acetone, which has fully removed any lipids.

**Figure 6:**
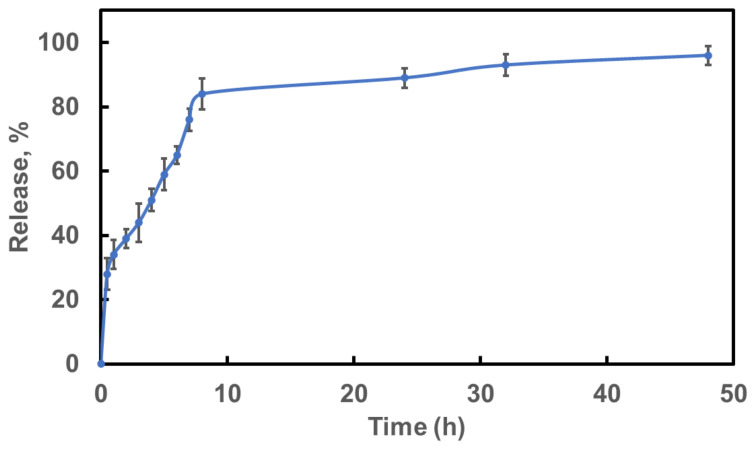
Permethrin release from the drug-halloysite composite in ethanol.

We estimated the wax content in capybara pristine hair and unwaxed hair using thermogravimetric analysis (TGA). A slight weight loss difference at the end of the 600 °C is attributed to the thermal decomposition of the wax, which constitutes ca 5 wt% of the pristine capybara hair (Figure 5D). This wax may be degraded in the region of 120 to 170 °C with the cortical cells denaturation which starts from 400 °C and the keratin denaturing which starts from 170 °C. Keratin fully degraded at 400–500 °C [32] and then the hair strands weight stabilized, as we show in Figure 5E. Using thermolysis, we also evaluated the loading of permethrin halloysite nanotubes. TGA profiles were obtained individually for permethrin, halloysite, and permethrin-loaded halloysite composites at a temperature ranging from 100–600 °C. Permethrin degrades within the temperature range of 200–430 °C. A peak at 480–520 °C indicates pristine halloysite degradation. Analysis of permethrin-halloysite composite quantifies 4.9 ± 0.5 wt% loading of drugs in the clay nanotubes which were used further for animal hair coating for the anti-lice experiment. The drug release profile from halloysite nanotubes was obtained in ethanol to demonstrate the maximum release of the drug over time. Permethrin release from the halloysites in ethanol was monitored for up to 48 h. Figure 6 shows an initial burst of the drug in ethanol within half an hour but after the first hour permethrin releases at a sustained rate with 86% released after 8 h and 98% release after 32 h. In a real scenario, the drug will be released in skin oil and water on the animal’s body which will give a much slower release than in ethanol, probably reaching many days and would be explored further in future studies.

### 3.3. Zeta Potential and Colloidal Stability of Halloysites

The colloidal stability of permethrin, permethrin unloaded, and loaded halloysites were assessed by zeta potential analysis in DI water for 3 days at room temperature. The zeta potential value of permethrin dispersion was found to be −37 mV on the first day and declined to −20 mV on the third day. In the same way, unloaded halloysite dispersion exhibited a zeta potential of −25 mV on day 1 and −3 mV on day 3 resulting in an essential sedimentation. On the other hand, the loaded halloysite dispersion showed −33 mV on day 1 and reached −20 mV on day 5 demonstrating good stability of the colloid.

### 3.4. Goat Hair Pretreated with Wax Followed by Halloysite Coating for Anti-Lice Protection

**Figure 7:**
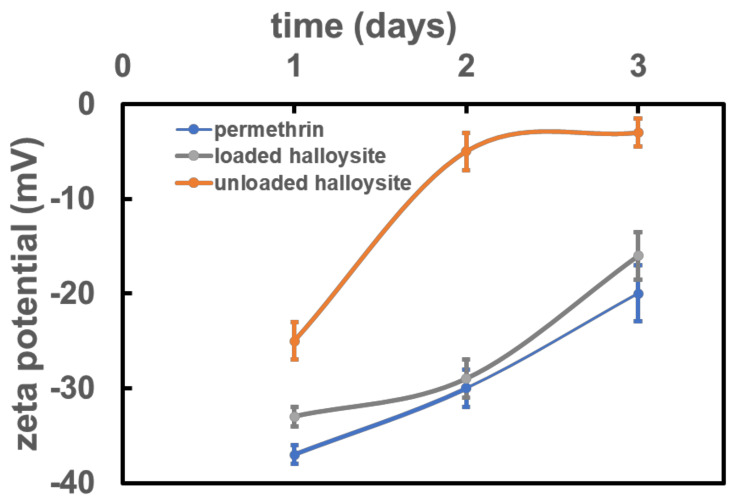
Zeta potentials of permethrin unloaded (pristine), and permethrin-loaded halloysites in water at pH 7.

Capybara hair, coated naturally with a wax layer, allowed for the fabrication of a denser and more robust halloysite coating as compared with non-waxed land-living animals, like guinea pigs and goats. This has stimulated us to apply a procedure for hair pre-treatment with diluted wax before coating with halloysite. This process gives increased coverage of halloysite coating on 0.1 wt% wax pre-treated hair surface allowing for 95% coverage. This halloysite coating can retain six shampoo washings thus demonstrating excellent stability and resilience to water exposure [3]. A diluted wax pretreatment drastically improved the hair nanoclay coverage, which is promising for ectoparasitic or fungal treatment. Figure 7 shows the pristine goat hair and goat hair treated by wax and coated with permethrin-loaded halloysite nanotubes. Wax pretreated hair drastically improves the coating and gave 100% halloysites coating on the pristine hair, with good coverage over cuticle gaps.

**Figure 8:**
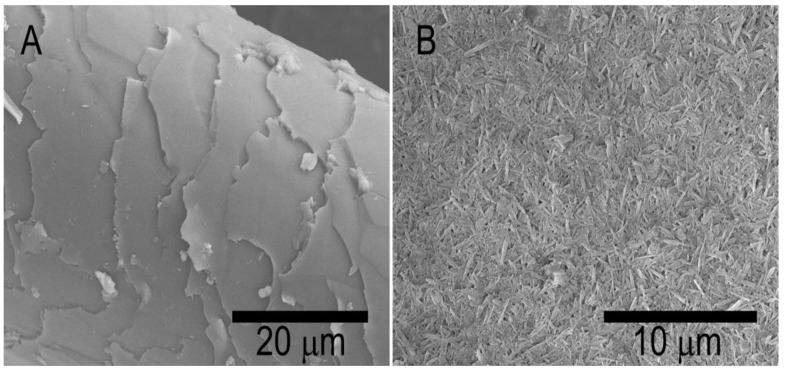
SEM images of pristine (**A**) and wax pretreated (**B**) goat hair coated with halloysite nanotubes.

Currently, a pour-on/spray-on permethrin treatment is available to control parasites on cattle [33], but its rain fast time is limited by several hours. The new halloysite-drug composites with aqueous dispersions give a long-lasting nano clay coating with sustained drug delivery on animal hair, and it is retained even after several water or shampoo washes or multiple rain showers, preventing the need for reapplication of the drug. Permethrin–halloysite formations were applied to the goat hair, providing a permanent stable coating (Figure 8) and such samples were exposed to live lice. The lice survival was monitored in comparison with current commercial treatment with permethrin solutions (used as a negative control).

### 3.5. Halloysite-Drug Formulation Antiparasite Efficiency on Goat Lice

**Figure 9:**
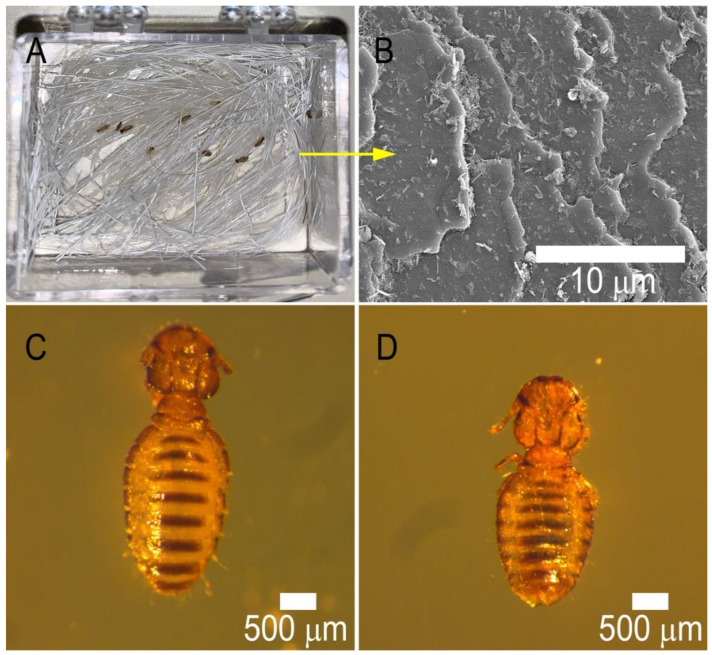
A photograph demonstrating the experimental setup and live lice crawling on halloysite-permethrin coated hair strands within the special humidity cell (glass box dimension 1 inch × 1 inch) (**A**) and SEM images of the same hair strand (**B**). Optical microscopy image of a young adult live louse (**C**) and a dead louse (**D**) with a noticeable curled body and shrunken abdomen.

The goal of this study was the development of an advanced technology to render animal hair with sustained anti-lice (antiparasitic) properties. We investigated the anti-lice effects using goat chewing lice *D. caprae*, which commonly infest goats (Figure 9). These species are closely related to other *Damalinia* genus representatives, such as *D. bovis*, therefore, the results obtained here can be extrapolated for ectoparasites that infect other species. First, we established the negative control effects using alcohol and pure halloysite (not loaded with permethrin). The results in Table 1 demonstrate the lack of any pediculicidal activity of pristine halloysite on goat chewing lice. All the samples were prepared with 30% aqueous ethanol and this concentration does not show any toxic effects on lice. This solvent is used in conventional permethrin solution for direct anti-lice formulations which we used as a control experiment. To check drug-free halloysite clay, hair treated with 5% and 10% HNT- alcohol dispersion was checked and showed no activity against the lice. This shows that the clay nanotubes and low concentration alcohol do not have any toxicity that could kill the insects (which has previously been demonstrated for other invertebrates [7], and the drug loading into halloysite is necessary for effective lice control).

Next, we established the efficiency of hair treatment with the drug in aqueous alcohol. To observe the effects of permethrin on mortality response on lice, 200 mg of goat hair was subjected to 0.80 mL of 0.5% permethrin in a 30% alcohol solution. Almost all the lice were dead within the first 4 h (Table 4). The dead lice were removed from this sample and the hair was rinsed with a tap water stream for 30 s. The hair was left to dry in the air. Then, a new batch of lice was reintroduced keeping the same amount of new skin flakes as food with the hair. Few lice survived within 4–8 h, and all the lice were dead in eight hours, showing that the drug remains effective. All the dead lice were removed from the sample, and the hair was washed for the second time with tap water for the next mortality check. After reintroducing 10 new lice in the damp hair with new skin debris as food, at least 6–7 species survived after 24 h. This indicates that almost all the drug was washed from the hair, and re-infestation of new lice cannot be prevented. Therefore, one traditional permethrin solution treatment will be hardly efficient after the animal is twice wetted with rain, and a new drug application is needed to prevent further lice infestation.

**Figure 10:**
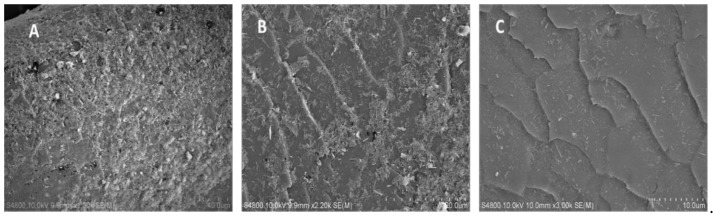
SEM images of drug-HNT composite deposited on goat hair (no washing) (**A**), hair washed one time after the clay deposition (**B**), and hair washed two times after deposition (**C**).

Finally, the long-term efficiency of the halloysite-permethrin coating after two hair washings over the time of a month was studied. The outcome was drastically improved with this hair coating of halloysite (HNT) loaded with permethrin, providing long-lasting sustainable anti-parasite protection. HNTs-permethrin coating pediculicidal activity was tested for goat hair treated with 0.8 mL of 5% HNTs-drug composite dispersion. The halloysite coverage on hair was confirmed using SEM (Figure 10A) where 70% of the surface was covered with loaded nanotubes. After four hours, two–three lice survived, and after 8 h, all the lice were dead (Table 5). We observed a delayed insecticidal activity because of the slower drug release from the nanotubes. We assume that the mortality could be kept at even higher rates if the permethrin molecules were also attached to the outer surface of the nanotubes by skipping the washing process after drug loading. After the death of all the lice in this first experiment, the hair was rinsed with a tap water stream and let dry, then a new batch of 10 lice was introduced on the samples with fresh food. The formulation efficiency was slightly decreased, and one–two lice survived after 4 h. The lice died slower this time because after rinsing, some halloysites were washed away while the drug was also releasing in an aqueous environment from the nanotubes. Figure 10B shows a halloysite coverage of approximately 50% after a second hair wash, which corresponds well with typical halloysite self-assembly patterns [34].

In a separate set of experiments, we removed all the dead lice, washed hair again, and reintroduced a new batch of lice; the treatment still worked well: two–three lice survived in 8 h and all the lice were killed within 16 h. The halloysite coverage on hair was observed in Figure 10C, where at least 15% of hair was covered with nanotubes. When the mortality results were compared with permethrin solution treatment after two hair washes, one can see a great improvement in the lice elimination. This implies that a stable water-resistant halloysite coating provides a sufficient drug amount for long-lasting animal protection.

We also performed a re-infestation check on halloysite-drug composite coated goat hair. According to the commercially available anti-parasitic drug, it must control the parasites for up to two months if not washed out. We reintroduced lice on the hair sample treated with traditional permethrin solution and coated it with HNTs-permethrin after four weeks to check the effectiveness. After this long-time exposure, the mortality rate and HNTs-drug formulation efficiency were preserved, while for a traditional treatment it was ended (Table S1). This implies that our halloysite-drug treatment is working well even one month after the topical application.

## 4. Conclusions

We elaborated an animal fur coating with 2–3 µm layer of halloysite nanotubes via spontaneous self-assembly from 5 wt% nano clay aqueous dispersion. Comparison of the selected water-living and land-dwelling animal’s hair coating with halloysite delineated the advantage of enhanced fur hydrophobicity with increased wax content in water-living capybara. This finding was used to improve the nanotube’s hair coverage for farm animals, such as goats, by simple pre-treatment with diluted wax. The nano clay coverage on the pretreated hair/fur of land-dwelling animals was protected for multiple washing, assuming stability against multiple rain wettings.

These clay nanotubes were loaded at 6 wt% with the anti-lice drug permethrin which showed slow release. For water resistance and long-lasting pediculicidal animal protection, we assembled such nanoclay-permethrin formulations on goat hair. The coating of permethrin-loaded halloysite on goat hair demonstrated efficient anti-lice protection for over one month even after two washing cycles. The direct drug treatment from alcohol solutions was terminated after the first animal washing because the animals lost antiparasitic protection. Such long-lasting topical hair/fur drug delivery may be applicable for other animal and human anti-lice treatments, as well as for psoriasis and fungal infection treatments.

We focused our research efforts on goat lice *D. caprae* from the Trichodectidae family, which do not consume blood as common human lice do. This species was used primarily for security reasons. However, we expect that the method reported here will be effective with other types of lice since all lice inhabit hair and keep close contact with the hair cuticle, regardless of dietary preference. Therefore, this approach is general and may be helpful for long-lasting and sustainable anti-lice protection, especially if a selection of insecticide drugs is encapsulated in addition to permethrin. These formulations also may be useful for the treatment of fur animals in zoological collections.

## Figures and Tables

**Table 1:** Microscale surface texture parameters in pristine and halloysite-treated hair, coated with halloysite (HNT). Sq (root mean square height if roughness); Sz (maximum roughness height); Ssk (Skewness); Sku (kurtosis-deviation from Gauss distribution; Sal (autocorrelation length); Str (texture aspect ratio); Sdr (developed interfacial area ratio).

Surface Parameter:	Sq (µm)	Sz (µm)	Ssk	Sal (µm)
Capybara untreated hair	1.5 ± 0.3	14.1 ± 5.3	−0.5 ± 0.5	9.8 ± 1.4
Capybara wax-removed hair	1.5 ± 0.1	10.9 ± 1.4	−0.62 ± 0.5	8.5 ± 2.1
Capybara HNTs coated hair	5.1 ± 3.8	33.3 ± 2.7	−0.7 ± 0.4	5.6 ± 1.4
Capybara wax-removed HNTs coated hair	2.2 ± 0.1	17.8 ± 0.7	−0.43 ± 0.1	3.4 ± 0.5
Guinea pig untreated hair	2.4 ± 0.3	25.3 ± 7.8	−0.3 ± 1.3	2.5 ± 2.4
Guinea pig HNTs-coated hair	4.1 ± 1.1	49.6 ± 2.5	−0.6 ± 1.0	1.7 ± 0.9

**Table 2:** Capybara and guinea pig hair cuticle AFM surface roughness and mechanical properties.

Parameter	Rq (nm)	Ra (nm)	Adhesion (nN)	Young’s Modulus (MPa)
Capybara (waxed)	171 ± 41	130 ± 31	5.3 ± 1.5	9.5 ± 1.9
Capybara (wax-free)	403 ± 59	314 ± 42	2.8 ± 1.7	12.4 ± 0.8
Capybara + HNT	137 ± 36	105 ± 26	7.4 ± 1.3	139.4 ± 38.6
Guinea pig	124 ± 22	100 ± 21	18.6 ± 3.2	63.5 ± 10.5
Guinea pig + HNT	115 ± 18	92 ± 10	8.6 ± 2.2	61.4 ± 26.2

**Table 3:** Negative control performance of 30% alcohol and halloysites (HNT) on *D. caprae* lice.

Experimental Formulation	Number of Lice Survived/Recorded Time (h)				
	0	4	8	16	24
Hair without any treatment	10	10	10	10	10 ± 1
Hair washed with 30% ethanol	10	10	10	10	10 ± 1
Hair coated with 5% unloaded HNT	10	10	10	10	10 ± 1
Hair coated with 10% unloaded HNT	10	10	10	10	10 ± 1

**Table 4:** Mortality of permethrin solution on the application on *D. caprae* lice after multiple hair washing.

Experimental Formulation.	Number of Lice Survived/Recorded Time (h)				
	0	4	8	16	24
Hair treated with 0.5% permethrin	10	1 ± 1	0	0	0
Hair washed one time after 0.5% permethrin treatment	10	5 ± 1	2 ± 1	0	0
Hair washed two times after 0.5% permethrin treatment	10	8	6 ± 1	6 ± 1	6 ± 1

**Table 5:** Mortality response of permethrin-halloysite (HNT) composites on *D. caprae* lice with first and second hair wash.

Experimental Formulation	Number of Lice Survived/Time (h)				
	0	4	8	16	24
Hair coated with permethrin—HNT, no washing	10	2 ± 1	0	0	0
Hair washed one time after permethrin-HNT treatment	10	3 ± 1	1 ± 1	0	0
Hair washed two times after permethrin-HNT treatment	10	4 ± 1	3 ± 1	0	0

## Data Availability

The data presented in this study are available on reasonable request from the corresponding author.

## Supplementary Materials

The following are available online at https://www.mdpi.com/article/10.3390/pharmaceutics13091477/s1, Figure S1: Self-assembly of halloysite clay nanotubes on acetone-washed (unwaxed) capybara hair surface, Figure S2: Cross-section image of halloysite coated pristine (A) and unwaxed (B) capybara hair-halloysites visible at the edges, Figure S3: SEM cross-sectional image of halloysite coated guinea pig hair, halloysite tubes (arrowed) are visible at the edges, Figure S4: SEM images of pristine horse hair (A) and partially halloysite coated (B) horse hair, Figure S5: Confocal optical images of the pristine goat hair (A) and wax pre-treated goat hair coated with permethrin loaded halloysite, Figure S6: AFM images of pristine capybara (A–C) and guinea pig (D–F) hair, Figure S7: Nanomechanical characteristics (adhesion, modulus) of the capybara and guinea pig hair surface before and after coating with halloysite nanotubes, Table S1: Mortality response of drug—HNT treatment after one month after 1st and 2nd hair wash.
