# Supplementary material for: Clay Nanotube Immobilization on Animal Hair for Sustained Anti-Lice Protection
Source: Pharmaceutics. 2021 Sep 15;13(9):1477. doi: 10.3390/pharmaceutics13091477 (PMC8466176; doi:10.3390/pharmaceutics13091477)
Supplement: Supplementary file 1 [file pharmaceutics-13-01477-s001.zip › pharmaceutics-1330220-supplementary.pdf]

pharmaceutics

Supplementary Materials: Clay Nanotube Immobilization on Animal Hair for Sustained Antilice Protection

Naureen Rahman, Faith Hannah Scott, Yuri Lvov, Anna Stavitskaya, Farida Akhatova, Svetlana Konnova, Gölnur Fakhrullina, Rawil Fakhrullin

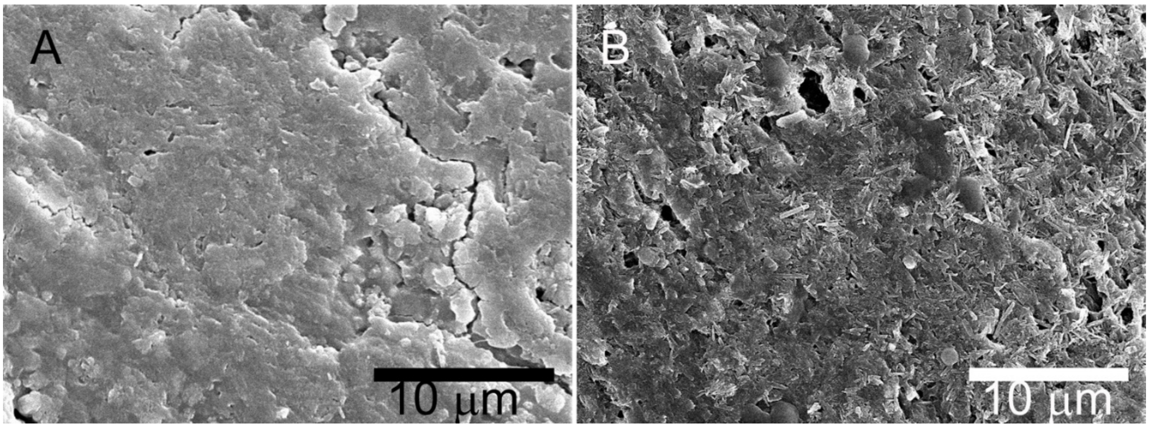

Figure S1. Self-assembly of halloysite clay nanotubes on acetone-washed (unwaxed) capybara hair surface: SEM images of the unwaxed capybara hair (**A**) and the surface is partially covered with HNTs (**B**).

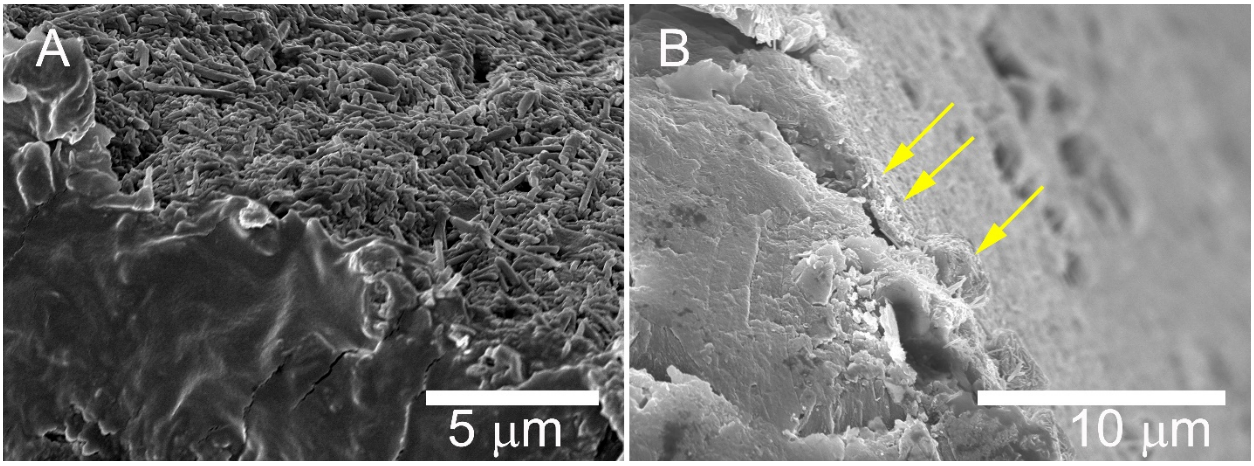

Figure S2. Cross-section image of halloysite coated pristine (**A**) and unwaxed (**B**) capybara hair-halloysites visible at the edges.

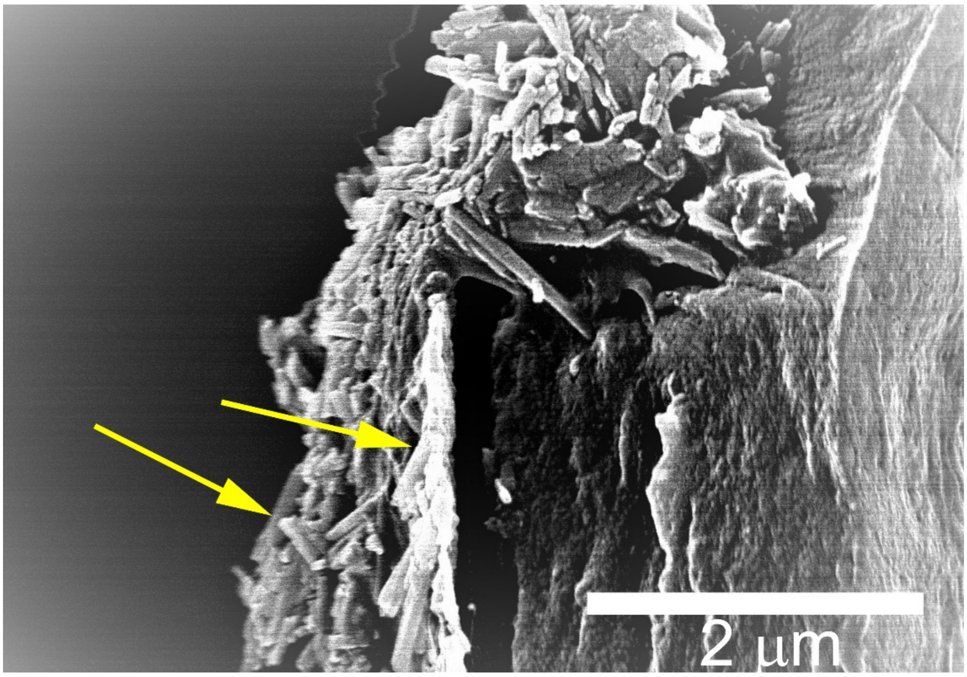

Figure S3. SEM cross-sectional image of halloysite coated guinea pig hair, halloysite tubes (arrowed) are visible at the edges.

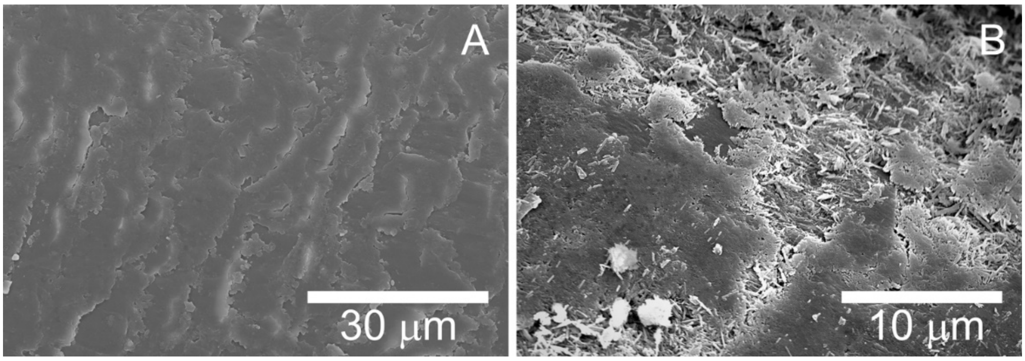

Figure S4. SEM images of pristine horse hair (**A**) and partially halloysite coated (**B**) horse hair.

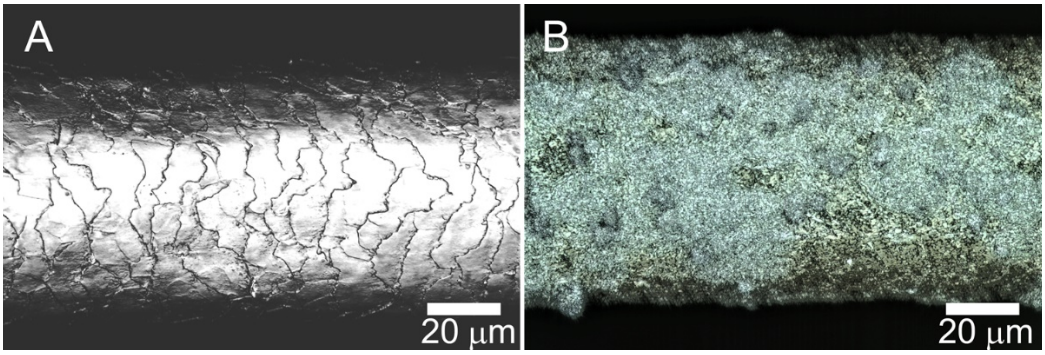

Figure S5. Confocal optical images of the pristine goat hair (**A**) and wax pre-treated goat hair coated with permethrin loaded halloysite (**B**).

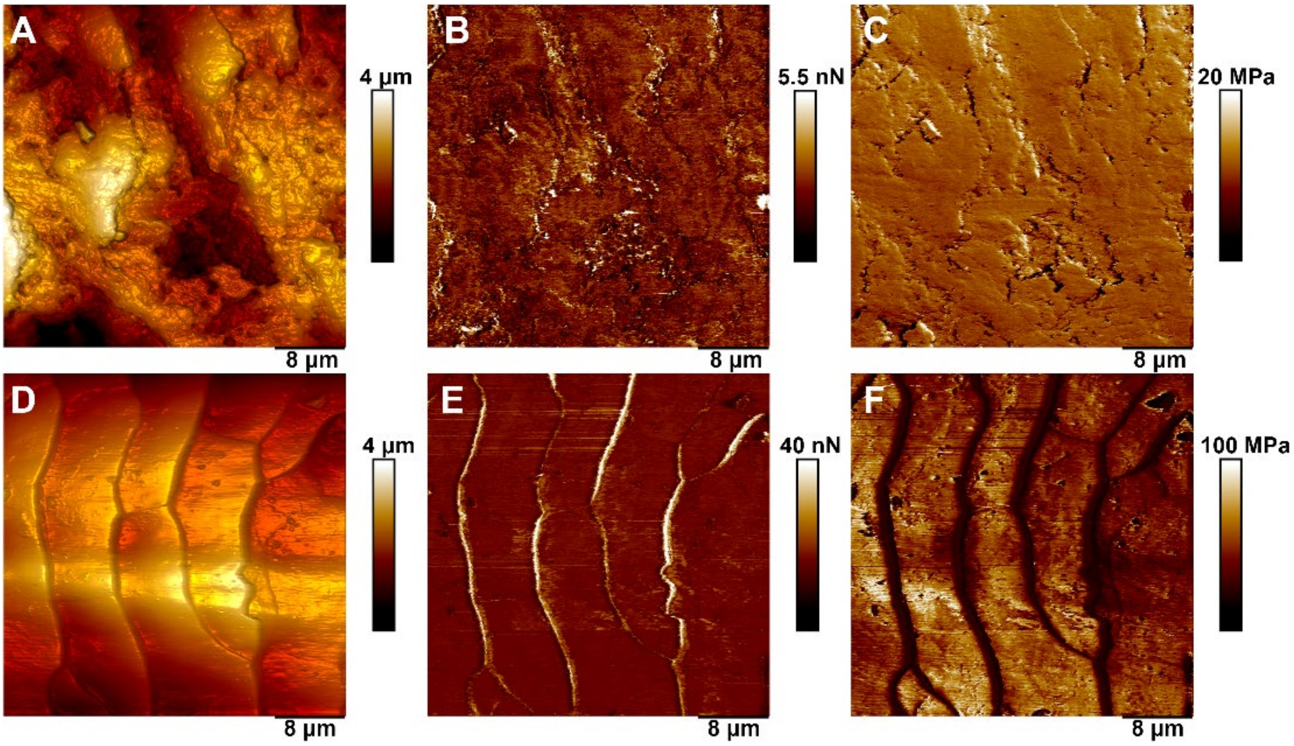

Figure S6. AFM images of pristine capybara (**A**–**C**) and guinea pig (**D**–**F**) hair: topography (**A**, **D**); adhesion (**B**, **E**) and Young's modulus map (**C**, **F**).

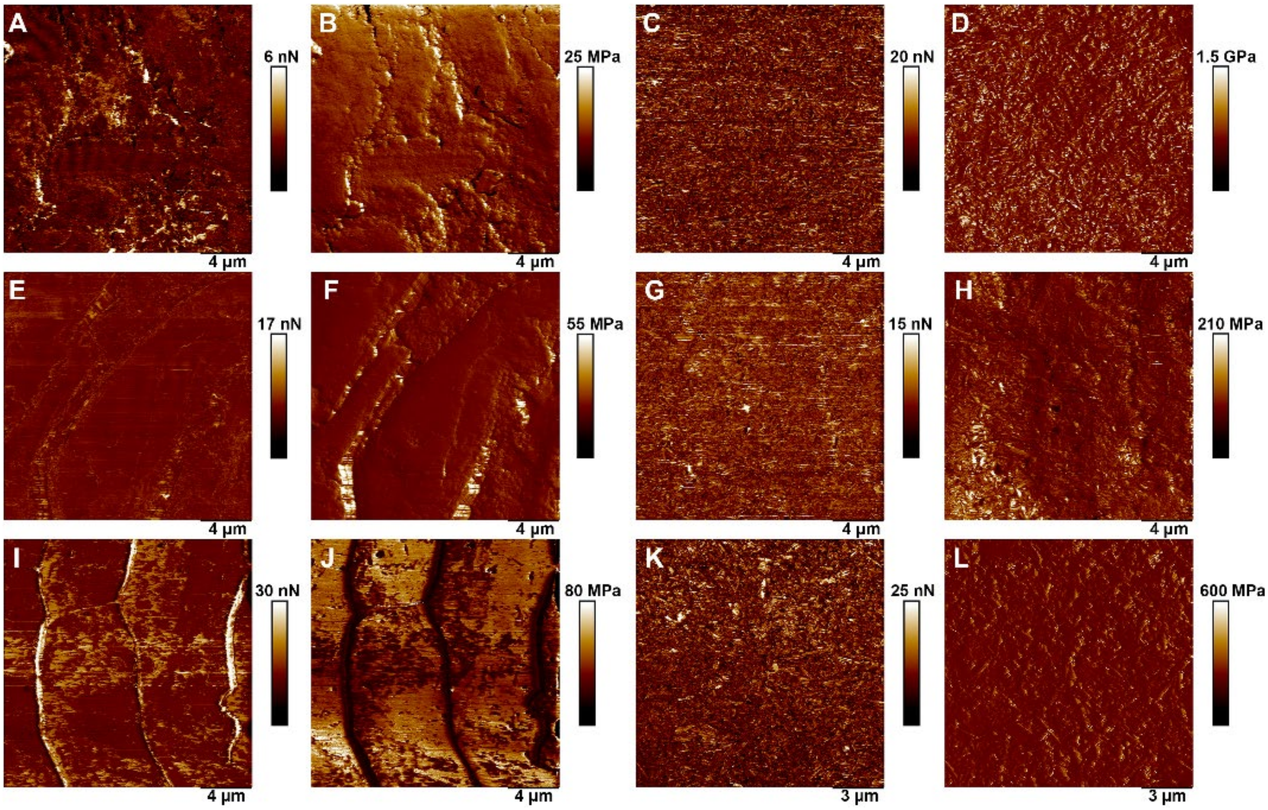

Figure S7. Nanomechanical characteristics (adhesion, modulus) of the capybara and guinea pig hair surface before and after coating with halloysite nanotubes: (**A**–**B**) pristine capybara hair; (**E**–**H**) unwaxed capybara hair; (**I**–**L**) guinea pig hair; (**A**, **E**, **I**) adhesion and (**B**, **F**, **J**) Young's modulus of halloysite-free capybara and guinea pig hair; (**C**, **G**, **K**) adhesion and (**D**, **H**, **L**) Young's modulus of halloysite-coated halloysite capybara and guinea pig hair.

Table S1. Mortality response of drug - HNT treatment after one month after 1st and 2nd hair wash.

**Experimental formulation**	**No of lice survived \ Time (h)**				
	**0**	**4**	**8**	**16**	**24**
Hair treated with 0.5 % permethrin drug	10	1 ± 1	1 ± 1	0	0
Hair washed one time after 0.5 % permethrin treatment	10	5 ± 2	2 ± 1	0	0
Hair washed two times after 0.5 % permethrin treatment	10	7 ± 1	7 ± 1	6 ± 1	6 ± 1
Hair coated with permethrin - HNTs, no washing	10	2 ± 1	2 ± 1	0	0
Hair washed one time after permethrin-HNTs treatment	10	5	2 ± 1	0	0
Hair washed two times after permethrin-HNTs treatment	10	6	3 ± 1	1 ± 1	0
